# Nutritional Regulation of Hepatic FGF21 by Dietary Restriction of Methionine

**DOI:** 10.3389/fendo.2021.773975

**Published:** 2021-11-30

**Authors:** Han Fang, Kirsten P. Stone, Laura A. Forney, Desiree Wanders, Thomas W. Gettys

**Affiliations:** ^1^Laboratory of Nutrient Sensing and Adipocyte Signaling, Pennington Biomedical Research Center, Baton Rouge, LA, United States; ^2^Department of Kinesiology, Houston Baptist University, Houston, TX, United States; ^3^Department of Nutrition, Georgia State University, Atlanta, GA, United States

**Keywords:** methionine restriction, protein restriction, energy expenditure, essential amino acids (EAA), nutrient sensing mechanisms

## Abstract

FGF21 is a potent metabolic regulator of energy balance, body composition, lipid metabolism, and glucose homeostasis. Initial studies reported that it was increased by fasting and the associated increase in ketones, but more recent work points to the importance of dietary protein and sensing of essential amino acids in FGF21 regulation. For example, dietary restriction of methionine produces a rapid transcriptional activation of hepatic FGF21 that results in a persistent 5- to 10-fold increase in serum FGF21. Although FGF21 is a component of a complex transcriptional program activated by methionine restriction (MR), loss-of-function studies show that FGF21 is an essential mediator of the resulting effects of the MR diet on energy balance, remodeling of adipose tissue, and enhancement of insulin sensitivity. These studies also show that FGF21 signaling in the brain is required for the MR diet-induced increase in energy expenditure (EE) and reduction of adiposity. Collectively, the evidence supports the view that the liver functions as a sentinel to detect and respond to changes in dietary amino acid composition, and that the resulting mobilization of hepatic FGF21 is a key element of the homeostatic response. These findings raise the interesting possibility that therapeutic diets could be developed that produce sustained, biologically effective increases in FGF21 by nutritionally modulating its transcription and release.

## Overview

The mammalian Fibroblast growth factors (*Fgf*) include an intracellular subfamily (*Fgf11-14)*, a hormone-like subfamily (*Fgf15/19/21/23)*, and a canonical subfamily (*Fgf1-10, Fgf16-18*, and *Fgf20)* (1). The *Fgf* family arose from an ancestral *Fgf* gene (e.g., *Fgf13*) during vertebrate evolution through two distinct gene and genome duplication events (2, 3). The resulting expansion diversified the signaling capabilities of *Fgf* family members, transforming them into ubiquitous regulators of developmental and metabolic processes. Canonical FGFs act locally as paracrine agents while hormonal FGFs are released into the circulation and function in an endocrine manner. The initial recognition of canonical FGFs as growth factors came after their isolation from the pituitary (4, 5) and from conditioned liver cell media (6). The remaining FGFs were identified using degenerative primers and homology-based PCR in conjunction with searches of DNA databases for homologous sequences (7). Canonical FGFs act through heparin-binding sites that stabilize their binding to FGF receptors. In contrast, hormone-like FGFs acquired endocrine functions by losing the heparin binding requirement and acquiring the ability to bind to their receptors using βKlotho as a cofactor. FGF receptors are ubiquitously expressed across multiple tissues, but the expression of βKlotho is far more restricted. This provides a mechanism to limit the target tissues of endocrine FGFs to specific sites. Of particular interest is the endocrine FGF, *Fgf21*, which came into sharp focus after it was discovered and shown to have beneficial effects on energy balance, insulin signaling, glucose uptake, and lipid metabolism (8–11). These properties inspired great enthusiasm for development of FGF21-based therapies for treatment of metabolic disease. The goal of this minireview is to briefly summarize recent efforts in that arena, the obstacles encountered, and outline an alternative approach to obtaining the beneficial metabolic effects of FGF21 through chronic nutritional modulation of endogenous FGF21 expression.

## Biological Effects of Exogenous FGF21

FGF21 was identified in cDNA from mouse embryos, found to be highly expressed in mouse liver (12), and shown to enhance glucose uptake in 3T3-L1 adipocytes through an insulin-independent increase in *Glut1* expression (10). Administration of FGF21 to genetically obese rodents reduced their body weights, lowered blood glucose and plasma triglycerides, and reduced fasting insulin (10). These metabolic effects were recapitulated in transgenic *Fgf21*– overexpressing mice (10) and in C57BL/6J mice treated with FGF21 *via* osmotic minipumps (8). FGF21 increased food intake per unit body weight in these studies but was still able to reduce body weight because it simultaneously increased EE (8). FGF21 increased thermogenic gene expression in white adipose tissue while reducing lipogenic genes in the liver (8). These findings were extended to C57BL/6J mice fed a high fat diet where FGF21 also improved overall insulin sensitivity by decreasing hepatic glucose production and increasing insulin-dependent glucose uptake in adipose tissue (11). FGF21 produced these diverse effects through a combination of centrally-mediated effects on sympathetic outflow (13), peripherally-mediated effects on glucose uptake in adipose tissue (14–18), suppression of hepatic glucose production (11), and improvements in biomarkers of metabolic health that are secondary to FGF21-dependent reductions in adiposity. FGF21 also proved effective in non-human primates, as daily injections of the hormone in diabetic rhesus monkeys reproduced the beneficial metabolic effects of FGF21 observed in rodents (9). Collectively, these studies with pre-clinical models provided a compelling rationale for developing FGF21-based pharmacotherapies for metabolic disease.

## Translational Responses to FGF21 Analogs and Mimetics

The relatively short biological half-life of FGF21 (19) and its conformational instability in solution presented significant logistical impediments to using native FGF21 in the clinic (20). Efforts to re-engineer the FGF21 molecule to improve its formulation stability and biopharmaceutical properties have been successful and multiple labs have developed FGF21 analogs or receptor mimetics that are stable in solution, have long biological half-lives, and show minimal toxicology (21–25). In addition, pre-clinical studies with rodents and non-human primates have verified that several FGF21 analogs and mimetics successfully reproduce the full range of metabolic benefits produced by the native molecule (19, 20, 26–30). Many of the FGF21 analogs and mimetics have progressed to clinical trials in patients with type 2 diabetes [see review by (31)]. However, the primary end points of improved glycemic control have not been met, although significant reductions in circulating lipids, hepatic fat, and body weight were documented in two studies (27, 32). In studies of patients with non-alcoholic steatohepatitis (NASH), pegylated versions of FGF21 produced significant decreases in hepatic fat, markers of hepatic fibrosis, and liver injury (33, 34). Although the FGF21 analogs were generally well tolerated, one of the analogs increased blood pressure and heart rate and produced modest increases in circulating markers of bone resorption (27). The latter safety concern is consistent with pre-clinical findings showing that transgenic *Fgf21* mice had higher rates of bone resorption and lower rates of bone formation (35, 36). Another adverse effect was the generation of FGF21 antibodies caused by the immunogenicity of the FGF21 analog (33, 34). This could be a more pervasive problem if the engineered structures of other FGF21 analogs and mimetics are recognized as foreign by the immune system, particularly since their use is expected to involve chronic treatments. It is clear that the safety issues associated with the long-term use of these drugs will need to be thoroughly examined going forward. Equally concerning is whether there are fundamental differences in the way non-human primates and humans respond to FGF21 in terms of glucose lowering (31). Viewed collectively, it appears that these unresolved issues will delay implementation of FGF21-based mimetics for broad-based treatment of metabolic disease for now.

A potential solution to the obstacles encountered with injectable pharmacotherapies based on FGF21 mimetics is provided by an approach that involves chronically increasing endogenous FGF21 transcription and release from the liver. Discovery research over the last decade has shown that the liver functions as a sentinel to monitor and respond to changes in dietary composition, particularly the protein and amino acid content of the diet (37–39). Reductions in the overall protein content of the diet or the amounts of specific essential amino acids (EAA) produce a rapid transcriptional activation of the hepatic *Fgf21* gene, and the increased expression of FGF21 is maintained for as long as the experimental diet is consumed (40, 41). The reductions in dietary EAA content needed to transactivate *Fgf21* without also compromising growth and development occur within a narrow concentration range, but within these defined ranges the resulting increase in circulating FGF21 reproduces the full range of metabolic responses produced by treatment with exogenous FGF21 (42). Therefore, the development of therapeutic diets that produce the required degree of EAA restriction provides a potentially attractive approach to obtain the benefits of FGF21 biology through diet-induced modulation of its expression. The specific dietary modifications that produce these effects have been rigorously established in recent years and potential approaches to their implementation will be the subject of the current minireview.

## Physiological Regulation of FGF21 Expression

The physiological context in which FGF21 regulation was originally identified was fasting or starvation when the associated increases in fatty acids and ketones activated PPARα and increased transcription of the hepatic *Fgf21* gene (43, 44). Ketogenic diets were proposed as important regulators and FGF21 was originally dubbed as a starvation hormone (43–45). However, except under extreme conditions, the significance of fasting and ketones now seems relatively unimportant in regulation of human FGF21 (46–48). Given the impact of FGF21 on dysfunctional glucose and lipid metabolism, a concerted effort has been made to identify the key metabolic states, physiological signals, hormones, signaling pathways, and transcription factors responsible for regulation of *Fgf21* (49–60). *In vivo* studies have been complemented by extensive *in vitro* work to identify and dissect the sensing and signaling systems responsible for transcriptional activation of the *Fgf21* gene (53, 55, 61–65). While it is beyond the scope of this minireview to provide a detailed accounting of the many signaling systems providing regulatory input to the *Fgf21* gene, several excellent reviews are available (45, 62, 66–71). A particularly useful illustration of the regulatory complexity of *Fgf21* transcription was presented by Erickson and Moreau (66), who used bioinformatic tools to map the response elements contained within the 5’-flanking region of 4600 bp of the transcription start site for the human, mouse, and rat *Fgf21* gene. They identified multiple copies of binding sites for no fewer than 10 nuclear receptors and/or transcription factors in the promoters. In addition, multiple sensing and signaling systems provide input to each nuclear receptor and transcription factor so this map of response elements does not capture the true complexity of *Fgf21* regulation (66). For example, ER stress signals through PERK and eIF2α to increase transcriptional activation of *Fgf21* through ATF4 (56, 72), but sensing of essential amino acids through GCN2 and eIF2α can also signal through ATF4 to increase *Fgf21* expression (73). Viewed collectively, the physiological state of the liver at any point in time is providing a vast amount of regulatory input to the *Fgf21* gene. However, from a therapeutic viewpoint, the most important question is whether the prevailing rate of *Fgf21* transcription can be reset to a higher overall rate that chronically increases circulating FGF21 and produces the associated metabolic benefits.

## Dietary Regulation of FGF21 Expression

Dietary protein restriction and methionine restriction (MR) produce a comparable series of behavioral, physiological, biochemical, and transcriptional responses that result in a significant improvement in metabolic health [reviewed in (37, 38, 74–76)]. The responses include increased energy intake and expenditure, decreased adiposity, enhanced insulin sensitivity, and reduction in circulating and tissue lipids. A common model used to study protein restriction involves reducing the dietary concentration of casein from 20% to 5% (77). Dietary MR involves formulation of diets from elemental amino acids, reducing the methionine content from 0.86% to 0.17%, and eliminating cysteine. Both dietary regimens result in a similar reduction in methionine and cysteine intake (77, 78), and both diets increase expression and release of hepatic FGF21 (77, 79, 80). Rodent studies show that protein restriction increases circulating FGF21 within 24 h (77) while chronic protein restriction permanently increased FGF21, enhanced metabolic health, and extended lifespan (81). The mouse longevity study (81) used an integrative modeling approach called the Geometric Framework with groups of mice fed one of 25 diets that systematically varied the protein, carbohydrate, and fat content. They found that FGF21 was maximally elevated under low protein intakes and the increased FGF21 was strongly correlated with improvements in biomarkers of metabolic health (41). Dietary MR produces a comparable 5- to 10-fold increase in circulating FGF21 within 6 h of initiating the MR diet (72) and the increase in FGF21 is maintained for as long as the MR diet is consumed (80, 82, 83). Based on what is known about FGF21 biology and the responses of rodents to exogenous or overexpressed FGF21, it is attractive to speculate that transcriptional activation of hepatic *Fgf21* by protein restriction or MR provides the causative link to the metabolic phenotypes produced by each diet. This hypothesis has been systematically addressed using multiple loss of function approaches with each diet. The Morrison lab (77) established that FGF21 was the endocrine signal linking protein restriction to increased EE, and showed in later work that FGF21 was also necessary for the low protein diet to increase thermogenic gene expression in brown and white adipose tissue (84). Low protein diets also enhanced glucose homeostasis in mice and FGF21 was necessary for this effect (85). FGF21 signaling in the brain increases EE by increasing sympathetic nervous system outflow (13) and mice that lack either *Fgf21* or FGF21 signaling in the brain are unable to increase EE in response to protein restriction. Parallel studies of dietary MR have been conducted with *Fgf21^-/-^* mice and mice with ablation of central FGF21 signaling. In *Fgf21^-/-^* mice, the ability of dietary MR to increase EE and enhance thermogenic gene expression in brown adipose tissue (BAT) and white adipose tissue (WAT) was totally dependent on FGF21, while effects of dietary MR to reduce lipogenic gene expression in the liver did not require FGF21 (40). Deletion of βKlotho in the CNS also completely blocked the ability of dietary MR to increase EE and remodel adipose tissue (86), supporting the view that the increase in FGF21 produced by dietary MR acts primarily in the brain to affect energy balance. Viewed together, these findings make a compelling case that it is possible to obtain a rapidly deployed but long-lasting set of metabolic responses to FGF21 by diet-induced transcriptional activation of the hepatic gene. An important remaining question is whether therapeutic diets can be developed that produce chronic, biologically effective increases in FGF21 in a clinical setting that produce some or all beneficial metabolic effects attributed to the hormone.

## Paths to Implementation of Dietary MR to Chronically Increase FGF21

Casein restriction to 5% faithfully reproduces the full metabolic profile of dietary methionine restriction and restricts sulfur amino acids to a similar extent. Therefore, it seems likely that dietary casein restriction is producing most of its biological effects by limiting dietary methionine intake. An important implication of this conclusion is that it supports the feasibility of deriving the therapeutic benefits of dietary MR by restricting protein intake in conjunction with a careful accounting of methionine content of the various proteins that make up the diet. The most straightforward way to restrict methionine is to formulate amino acid-based diets that reduce the methionine content to ~0.17% and eliminate cystine. This is the approach used in pre-clinical studies of MR because rodents readily adapt to consumption of these diets. However, this approach works poorly in humans because of poor tolerance of the bitter, metallic taste of elemental amino acids. The medical food, Hominex^®^-2 is a methionine free mixture of essential amino acids that was developed to provide nutritional support to patients with pyridoxine-insensitive hypercystinuria or hypermethionemia (87). Although Hominex-2 was moderately effective in increasing fat oxidation and reducing hepatic lipid content in patients with metabolic syndrome, the high withdrawal rates from the study and subsequent feedback made it clear that poor palatability was a significant drawback (88). A second major drawback to the use of Hominex-2 is that it contains significant amounts of cystine, which effectively spares methionine and lessens the severity of the methionine restriction (89). As shown by several authors, the addition of even small amounts of cystine to MR diets effectively reverses the metabolic effects of MR (72, 90, 91). For example, in work using diets containing 0.17% methionine, the addition of 0.2% cystine completely reversed the ability of the 0.17% methionine diet to increase EE and reduce fat mass (72). Therefore, a critical question to be answered is how much dietary cystine can be present for a given amount of MR and still preserve the effects of the methionine restriction alone. In earlier work, the upper threshold of methionine restriction was ~0.25% methionine when no cystine was present in the diet (42). Recent work suggests that protein restriction producing reductions of methionine and cystine in the range of 0.24% to 0.26% retain full efficacy (78). Together these findings suggest that careful attention to the total amount of methionine and cystine will be needed to implement a protein-restricted diet that produces therapeutically effective reductions in sulfur amino acids.

Lastly, an alternative approach to dietary methionine restriction was recently described which reduced the methionine and cystine content of casein by targeted, oxidative deletion of the sulfur amino acids in the intact protein (92). The advantage of this approach is that the sulfur amino acid-depleted casein maintains its palatability and can produce the metabolic benefits of protein restriction without reducing overall protein content of the diet. Proof-of-concept studies comparing me-thionine-depleted casein-based diets to elemental amino acid-based methionine restricted diets established the feasibility of this approach and showed that the beneficial metabolic effects of methionine restriction were reproduced by the oxidized casein-based MR diets (92). It will be interesting in future studies to determine whether combinations of mild protein restriction coupled with targeted methionine depletion of the proteins to be restricted can be implemented to produce therapeutically effective diets for the treatment of obesity and metabolic disease.

## Conclusions and Future Directions

**Figure 1:**
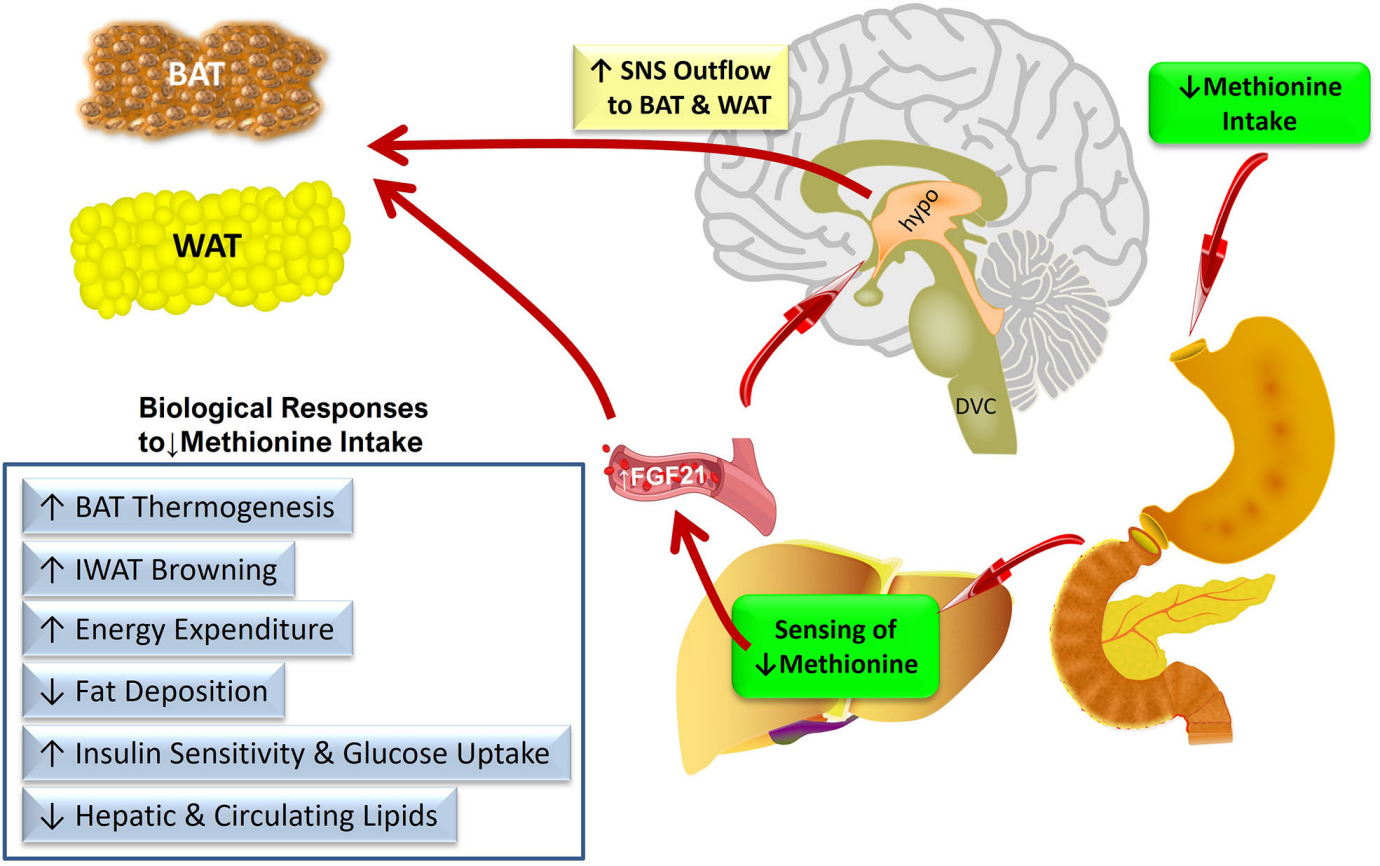
Conceptual model of the anatomical organization of the sensing and signaling systems that link increased transcription and release of FGF21 from the liver to the metabolic responses produced by dietary methionine restriction. Abbreviations used – BAT, brown adipose tissue; IWAT, inguinal white adipose tissue; SNS, sympathetic nervous system; DVC, dorso-vagal complex; hypo, hypothalamus.

**Figure 1** provides a conceptual model of the anatomical organization of the sensing and signaling systems that link increased transcription and release of FGF21 from the liver to the metabolic responses produced by dietary methionine restriction. We propose that FGF21 is the critical mediator of all the physiological responses to MR except its effects on hepatic lipid metabolism. An important remaining objective is to identify the sites where FGF21 is acting to produce the components of the overall biological response to dietary MR. For example, are the documented effects of dietary MR on insulin sensitivity in adipose tissue the result of direct actions of FGF21 signaling in this tissue, or are they secondary to the FGF21-mediated remodeling of adipose tissue that results from FGF21-dependent increases in SNS outflow to adipose tissue? These questions could be addressed in future studies using a tissue-specific loss of function approach that alternatively and selectively deletes FGF21 signaling in adipose tissue or the hypothalamus.

Although significant progress has been made in identifying specific cell types within the hypothalamus that respond to FGF21 (93–95), much additional work is needed to precisely identify the population(s) of neurons that link MR-dependent increases in FGF21 to the resulting biological responses.

Success in these experiments will guide the development of targeting vectors and the corresponding loss of function models that will be needed to provide definitive *in vivo* identification of the neurons linking MR-dependent increases in FGF21 to SNS activation. An additional challenging aspect of these experiments will be the interdependence of the multiple components of the phenotype (e.g., adiposity, insulin sensitivity) and correctly mapping the loss of MR-dependent responses to the anatomical site where FGF21 is acting to produce them.

The path to translational implementation of dietary MR using either protein restriction or targeted oxidation of methionine and cysteine in intact proteins will involve producing and testing the acceptability and efficacy of the resulting diets. A more practical path forward might be to use a combination of both approaches to develop a limited but highly palatable group of modified proteins that could be the basis for a therapeutic diet that is consumed under medical supervision for a specified interval. The successful implementation of dietary MR in a translational context will require the collaboration of food scientists to produce palatable modified protein, nutritionists to make sure methionine and cysteine are kept within the required range, and translational scientists to evaluate the safety and efficacy of the resulting diets.

## Author Contributions

HF, KS, LF, DW, and TG contributed to the writing and editing of the manuscript and agree to be held accountable for the content of the work. All authors contributed to the article and approved the submitted version.

## Funding

This work was supported by NIH DK-096311 (TWG). This work also made use of the Genomics Core Facility supported by NIH P20-GM103528 (TWG) and NIH 2P30 DK072476. This research project used the Transgenic and Animal Phenotyping core facilities that are supported in part by the NORC (NIH 2P30 DK072476) and by an equipment grant (S10OD023703) from the NIH. This work was also supported by NIH DK-098918; Center for Neuroinflammation and Cardiometabolic Diseases Seed Grant (DW).

## Conflict of Interest

The authors declare that the research was conducted in the absence of any commercial or financial relationships that could be construed as a potential conflict of interest.

## Publisher’s Note

All claims expressed in this article are solely those of the authors and do not necessarily represent those of their affiliated organizations, or those of the publisher, the editors and the reviewers. Any product that may be evaluated in this article, or claim that may be made by its manufacturer, is not guaranteed or endorsed by the publisher.
